# Neuro-Immune Abnormalities in Autism and Their Relationship with the Environment: A Variable Insult Model for Autism

**DOI:** 10.3389/fendo.2014.00029

**Published:** 2014-03-07

**Authors:** Daniel K. Goyal, Jaleel A. Miyan

**Affiliations:** ^1^Faculty of Life Sciences, The University of Manchester, Manchester, UK

**Keywords:** autism spectrum disorder, neuro-immune, environment, gut microbiota, neuroinflammation

## Abstract

Autism spectrum disorder (ASD) is a heterogeneous condition affecting an individual’s ability to communicate and socialize and often presents with repetitive movements or behaviors. It tends to be severe with less than 10% achieving independent living with a marked variation in the progression of the condition. To date, the literature supports a multifactorial model with the largest, most detailed twin study demonstrating strong environmental contribution to the development of the condition. Here, we present a brief review of the neurological, immunological, and autonomic abnormalities in ASD focusing on the causative roles of environmental agents and abnormal gut microbiota. We present a working hypothesis attempting to bring together the influence of environment on the abnormal neurological, immunological, and neuroimmunological functions and we explain in brief how such pathophysiology can lead to, and/or exacerbate ASD symptomatology. At present, there is a lack of consistent findings relating to the neurobiology of autism. Whilst we postulate such variable findings may reflect the marked heterogeneity in clinical presentation and as such the variable findings may be of pathophysiological relevance, more research into the neurobiology of autism is necessary before establishing a working hypothesis. Both the literature review and hypothesis presented here explore possible neurobiological explanations with an emphasis of environmental etiologies and are presented with this bias.

## Introduction

Autism spectrum disorder (ASD) is a neurodevelopmental disorder of unknown etiology. Recent evidence suggests a strong environmental component (1) and persistent neuroinflammation (2–5). Within the phenology of ASD and associated disorders, the subjectivity involved in attributing an infant or toddler with introversion (or being in one’s own world, autism) is fraught with difficulty. The difficulty is not whether such behavioral abnormalities represent a neurobiological illness – consensus is for an organic brain disorder – the challenge stems from the wide-ranging possibilities underlying the visible disease (6, 7). A secondary obstacle to the adequate identification of disease process in ASD patients pertains to scientific disparity. Research efforts have focused more on the genetic aspects of ASD than on environmental factors over the previous 15 years (8). Consequently, several important factors have impinged upon progress for ASD sufferers and those at risk.

The fixation on genetics led to reprioritization by medical staff and displacement of non-genetic scientific researchers. Many clinicians awaited the genetic answer and the promise of targeted, scientifically originated treatment. Conveying the certainty of the scientific consensus at the time, clinicians are now faced with the same patients and a different, almost polar growing certainty: there are likely to be prognostic factors one can mitigate (9, 10).

Previous twin studies suggested a predominant genetic component; however, these studies were poorly designed and had weak power (16, 17). A recent twin study published in July 2011 was well-designed with a substantial statistical power. One hundred ninety-four twin groups were studied and clinically evaluated prior to statistical analysis. Probandwise concordance for monozygotic twins was 77% (95% CI, 65–86%) for 45 male pairs and 50% (95% CI, 16–84%) for 9 female pairs. Concordance rates for dizygotic twins were 31% (95% CI, 16–46%) and 36% (95% CI, 11–60%) for 45 male and 13 female pairs, respectively. The study concluded: autism has substantial environmental factors, and indeed the environmental factors were of more significance than genetic factors (1).

There has been another compounding factor. Diagnostic labeling has changed substantially. Since its discovery in the late 1930s, autism has gradually become the diagnosis of choice. It has replaced and superseded childhood schizophrenia and feeble-mindedness and has encompassed within the spectrum, a host of neurodevelopmental disorders [for review see Ref. (11)]. This allowed a rational argument for the increase in prevalence, and as such a tempering of the strict scientific critique required.

Recent advancements in ASD research has led to a surge in research activity, in particular neuro-immune and environmental factors. Here, we present a view of ASD related to neurological, immunological, and neuroimmunological findings from the bias of an environmental etiology standpoint. We briefly discuss the pertinent literature concerning the frequently reported abnormal gut microflora composition in ASD patients. Finally, based on the growing consensus in biological scientific evidence and clinical experience, we present the variable insult model of ASD with the aim of contributing further to a useful research direction for those suffering from ASD and for those faced with managing the condition.

## Epidemiology

Autism spectrum disorder was first identified by Kanner in 1938 (12). Over the subsequent 10 years, Kanner discovered 50 further cases (13). Kanner subsequently reviewed the first 11 patients at 30-year follow-up. Only one known patient achieved employment (14). More recent evidence also suggests a high level of disability in affected individuals, with 60–75% achieving poor or very poor outcomes in adulthood (15).

Autism spectrum disorder case detection rates are now substantially higher – from 1 in 3000 reported in 1966 (including both autism and psychosis) (16), 1 in 150 in 8-year-olds in 2007 [Centre for Disease Control (CDC), (17)], and in 2012 a rate of 1 in 88 [CDC, (18)]. In UK, Cohen et al. described a prevalence rate of 1 in 64 (19).

## Morbidity and Mortality

Shavelle et al. investigated the mortality rate of ASD in over 13,000 patients between 1983 and 1997 (20) and found it to be more than twice that of neurotypical peers. Standardized mortality ratio (SMR) was estimated as 2.4. Certain causes carried significantly higher SMR (see Table 1). Similar mortality rates have been reported in other studies (21, 22) with a consistent increased mortality rate for ASD, and a substantially greater risk in female ASD patients. Whilst mental retardation predicted risk of early demise, those without mental retardation also had increased risk.

**Table 1:** **Causes of death in ASD with moderate to severe retardation or none to mild retardation (in brackets)**.

Cause of death	Early childhood SMR (5–10 years)	Late childhood SMR (10–20 years)	Adulthood SMR (>20 years)
Drowning	90.6 (14.1)	n/s	n/s
Digestive	n/s	40.8	5.9
Respiratory	n/s	24.5	9.4
Cancer	n/s	12.0 (3.8)	2.4 (1.6)
Nervous and sense	n/s	6.4 (15.9)	4.1
Seizures	n/s	n/s	30.8 (33.1)
Cardiovascular	n/s	n/s	3.7 (2.2)

*Adapted from Ref. (20). SMR, standardized mortality ratio; n/s, no significant increase in SMR found in either group*.

## Disease Progression

There have been several studies evaluating diagnostic stability over time. Turner et al. reassessed 2-year-olds diagnosed with ASD at 4.5 years of age (23) and found no change in their diagnosis of ASD but did find that 20% of children worsened between 2 years of age and 4.5 years of age and 20% improved. Within the parameters addressed, 60% remained relatively stable. No reason was identified for the variation.

Levy et al. have recently reviewed the literature regarding long-term outcome in ASD finding cognitive improvement in 20–55%, cognitive stability in 20–70%, and cognitive loss in 10–15% (24). No reasons were identified for why some ASD patients suffer a progressive illness and others make some recovery. ASDs as a group carry a poorer prognosis than other developmental disorders in almost all domains (24).

## Summary of Epidemiological Findings

Even though ASD is associated with high health, social, and financial impacts, investigative epidemiology has been limited. Perhaps the premature acceptance of ASD as a genetic condition limited the power of epidemiological science beyond that of detection of cases [for review see Ref. (25)]. The Interagency Autism Coordinating Committee (IACC) and Centers for Autism and Developmental Disabilities Research and Epidemiology (CADDRE) Network are co-ordinating a large epidemiological study in the US: the study to explore early development (SEED) (26). This is in keeping with the responsibilities set out in the US through “The Combating Autism Act 2006.”

## Immune Abnormalities and Neuroinflammation in ASD

Perhaps one of the most substantive studies in the last decade was conducted at the John Hopkins Institute, and involved an analysis of autopsy specimens and cerebrospinal fluid (CSF) samples from affected individuals and controls (2). The results indicated a neuroinflammatory response, regardless of age (in patients between 5 and 46 years of age), involving excess microglial activation and increased pro-inflammatory cytokine profiles. The study carries high statistical significance [for review of study, see Ref. (27)] and indicates an inflammatory state probably exists in the brains of these patients. Similar findings were found in a more recent autopsy study of microglia densities in fronto-insular and visual cortices of patients with ASD versus controls, and found a statistically significant (*p* ≤ 0.0002) increase in microglial density in both regions (4). Other immune abnormalities have also been found indicating an inflammatory state. Transforming growth factor beta 1 (TGF-β1) is reduced in ASD cohorts versus controls and individuals with other developmental disorders and was found to be inversely proportional to behavior outcomes (irritability, lethargy, stereotypy, and hyperactivity) as well as with levels of social adaptability (28).

Natural killer cells (NK cells) are abnormal in sub-groups of ASD. NK cells respond to macrophage-derived cytokines and are essential in tumor prevention and host anti-viral activity. Enstrom et al. (29) found a significant reduction in NK cell cytotoxicity and a 2.5-fold increase in KSP-37, an NK gene normally induced during active viral infection. They concluded that ASD patients have activated but resting NK cells with increased levels of cytolytic proteins and an altered response to stimulation with changes in gene expression (29). Supporting these findings, cancer mortality rates are higher in ASD (20), and the only identified risk factor for mortality associated with the recent H1N1 outbreak was developmental delay (30). Both of these findings suggest immune dysfunction in ASD, and either or both of these findings could be linked with the NK cell abnormalities identified by Enstrom et al. (29).

There have been studies making correlations between measures of immune functions and cytokine profiles with behavioral measures in ASD (Table 2). Significant correlations were shown between certain behavioral indices and the chemokine’s, macrophage chemoattractant MCP-1, macrophage inflammatory protein (MIP)-1β, eotaxin, and “regulated upon activation normal T-cell expressed and secreted” factor (RANTES) (31). RANTES was associated with lethargy, stereotypy, and hyperactivity. Eotaxin was associated with hyperactivity, visual perception, fine motor control, expressive language, communication and daily living skills, and socialization. MCP-1 was associated with visual perception. These associations, if proven to be functional, raise many questions pertaining to the immune system’s connectivity to the nervous system and involvement in neurobehavioral illnesses (for summary of immunological findings relating to behavior in ASD, see Table 2). Of importance here is the probability of immune involvement in the core features of ASD. These findings also raise the possibility of assessing behavioral changes in ASD through a quantitative measure.

**Table 2:** **Behavior and immune functions in ASD [adapted from Ref. (84)]**.

Studies	*n*	Age	Assessment method	Immune measure	Behavior measure
Ashwood et al. (28)	143	2–5	ADI-R, ADOS, SCQ, VABS, MSEL, and ABC	Plasma levels of active TGFβ1	Lower TGFβ1 levels were associated with lower adaptive behaviors and worse behavioral symptoms
Iwata et al. (89)	37	20–25	ADI-R	Plasma levels of P-selectin	Lower levels of P-selectin associated with poor social development
Heuer et al. (90)	271	2–5	ADI-R, ADOS, and ABC	IgG levels in plasma	Decreased IgG associated with increased aberrant behaviors
Grigorenko et al. (91)	1059	n/s	ADI-R and ADOS	Genotyping of the MIF gene and plasma levels of MIF (*n* = 20)	Plasma MIF levels were positively correlated with worse scores on ADOS for social impairment and imaginative skills
Onore et al. (92)	60	2–5	ADOS, ADI-R, MSEL, VABS, and ABC	Induced cytokine response to PHA	Negative correlation between PHA induced IL-23 production and sociability scores of the ADOS
Enstrom et al. (93)	30	2–5	ADI-R, ADOS, SCQ, VABS, MSEL, and ABC	Monocyte TLR ligand stimulation	More impaired social behaviors and non-verbal communication are associated with increased production of IL-1β and IL-6 after TLR4 stimulation
Ashwood et al. (94)	139	2–5	ADI-R, ADOS, SCQ, VABS, MSEL, and ABC	Induced cytokine response to PHA and LPS	Pro-inflammatory or TH1 cytokines were associated with greater impairments in core features of ASD as well as aberrant behaviors; GM-CSF and TH2 cytokines were associated with better cognitive and adaptive function
Goines et al. (95)	466	2–5	ADI-R, ADOS, SCQ, VABS, MSEL, and ABC	Antibodies directed against a 45 or 62 kDa cerebellum protein	Children with antibodies directed against a 45-kDa cerebellum protein had increased lethargy and stereotypy; children with antibodies against a 62-kDa cerebellum protein showed increased aberrant behaviors on the VABS composite standard score
Kajizuka et al. (96)	62	6–19	ADI-R	Serum levels of PDGF	Increased serum levels of PDGF-BB homodimers positively associated with increased restricted, repetitive, and stereotyped patterns of behavior and interests
Ashwood et al. (31)	175	2–5	ADI-R, ADOS, SCQ, VABS, MSEL, and ABC	Plasma chemokines CCL2, CCL5, and eotaxin	Plasma chemokine levels associated with higher aberrant behavior scores and more impaired developmental and adaptive function
Ashwood et al. (97)	223	2–5	ADI-R, ADOS, SCQ, VABS, MSEL, and ABC	Plasma levels of cytokines IL-1β, IL-6, IL-8, and IL-12p40	Elevated cytokine levels in plasma were associated with more impaired communication and aberrant behaviors
Ross et al. (98)	16	3-31	ADI-R	GM-CSF, INFγ, IL-12p70, IL-1β, IL-6, IL-8, TNFα, and IL-10	Elevation of cytokines correlated with autistic symptoms in patients with 22q11.2 deletion syndrome

## Neurological Abnormalities in ASD

With the exception of neuroinflammatory changes, most reported neurobiological abnormalities in ASD are inconsistent.

Structurally, abnormalities have been described in the cerebellum, hippocampus, amygdala, and insular cortex (32). Abnormal brain volume has also been identified [for review see Ref. (33)]. A meta-analysis reported on an average of 13% smaller brain volume at birth, an average of 10% larger brain volume at 1 year of age than controls, and 2% larger in adolescence (33). An increase in gray matter with a reduced unit density has been quite reliably identified in this cohort (33). CSF volume has also been reported to be increased with enlarged ventricles (34) and mini-columnar size is decreased (35). It has been proposed that such structural variation may affect adaptation and hence learning, and may account for the heterogeneity and wide-ranging functional deficits seen in ASD (36). Disordered neural connectivity has been discussed for some time (37). The evidence supports under-connectivity between sensory cortices and association cortices in moderate to severe ASD, essentially leading to a failure to assimilate sensory information into a working environmental context, and a lack of connectivity of associative cortices to the frontal cortex in higher functioning autism (38). This helps explain associated learning difficulties in low functioning autism, and the poor fine motor control and impaired imitation identified in higher functioning autism (39–41).

## Autonomic Dysfunction

Autonomic involvement in ASD has been widely reported for over 30 years (42–52). A recent controlled trial explored in detail the nature and type of autonomic involvement (49). Real-time variability together with continuous monitoring of blood pressure and breathing rhythms were assessed in an ASD cohort versus controls. Over 80% of the ASD cohorts were found to have a reduced vagal tone, highly suggestive of low central parasympathetic activity and, significantly, in a separate study, vagal tone in the neonate was found to predict neurodevelopmental outcome more accurately than birth weight, socio-economic status, or co-morbid medical conditions (50). Given that the autonomic nervous system (ANS) is responsible for the majority of sensory information received by the central nervous system, any disruption to the ANS is likely to have wide-ranging effects on higher cortical development. In a longitudinal follow-up study, Goytag et al. examined the order of cortical development using repeat MRI and concluded: higher-order association cortices mature only after lower-order somatosensory and visual cortices, the functions of which they integrate, are developed (53). The development of a normal parasympathetic tone is thus likely to be crucial for adequate neurodevelopment [for review see Ref. (50)]. More research is required.

## Environmental Aspects of ASD

Hertz-Picciotto et al. provide a detailed review of developmental immunotoxicity (DIT) in relation to neurodevelopment disorders (54). Xenobiotic exposure in early life may lead to altered immune function throughout life, a persistent neuroinflammatory response and systemic immune dysregulation, and the possibility of a neurobehavioral manifestation of the disease (54).

The type of immune dysfunction relates to the type of xenobiotic involved and the timing of xenobiotic exposure with dose-dependent effects not as applicable in the developing immune system but specific neurological development depends on signaling from the immune system (55). Such conveyance of environmental state to the nervous system from the immune system is advantageous and essential, but is incompatible with adequate neurodevelopment in poor environmental conditions. Whether there is a single type of xenobiotic involved in the etiology of ASD or whether the pathophysiology involves exposure to general environmental pollution remains a keenly researched area. Pesticides, flame retardants, heavy metals, traffic fumes, and endotoxins have all been implicated (56–61). The apparent lack of consistency is further compounded by difficulties in measuring chronic toxicity and toxin-induced disruption particularly if the xenobiotic exposure is no longer present.

Dietert et al. describes several similarities between early-life immune insults (ELII), including DIT and ASD with gender differences, time-windows for immune development, and the corresponding variable presentation in both ELII and ASD making a compelling argument (62). Studying DIT and indeed developmental neurotoxicity requires functional measures and a history of significant exposure. Complicating matters further is the modest, but significant genomic variation in xenobiotic metabolism and hence resistance or vulnerability to environmental exposures.

## Abnormalities in Gut Microflora in ASD

Abnormal clostridia species have been found repeatedly in ASD (63–67). The theory of clostridia involvement was postulated by Bolte in 1998 who suggested that clostridia toxin adversely affected neurotransmitter function that could result in neurobehavioral changes presenting as autism (68). Supporting this hypothesis, Parracho et al. outlined robust measures of microflora abnormalities in ASD cases suffering from bowel problems using PCR analysis and found a clear and consistent abnormality in the clostridia species present in ASD sufferers versus controls. *Clostridium histolytica* were found in higher levels in the ASD group versus healthy unrelated controls (*p* < 0.01) and healthy related controls (*p* < 0.05) (65).

A clinical trial was carried out to assess the bowel and behavioral impact of anti-microbial therapy directed against these abnormal clostridia species (69). Oral vancomycin was used for 6 weeks. Behavioral measurements were carried out before and after, as well as clinical assessment of bowel symptoms. The numbers were low but the response to intervention was reported as statistically significant. 8 of the 10 patients studied improved in terms of behavior and bowel symptoms with some scoring within the neurotypical range. Discontinuation of vancomycin after the 6-week trial period led to a gradual regression in bowel and behavioral symptoms in all participants (69) suggesting that gut environment gives preference to these abnormal species. As yet, there has been no investigation of the combined approach of anti-microbial therapy and other interventions targeted at altering microbiota composition.

Williams et al. recently reported consistently abnormal Firmicutes to Bacteriodetes ratios from biopsy specimens in children with ASD versus inflammatory bowel disease (IBD) controls. This was linked to reduced disaccharidases (starch digesting enzymes), which in the same study were also found to be low in the ASD group. Williams et al. postulated a link between high carbohydrate transit to the large intestine in ASD leading to alteration in the proportion of Firmicutes to Bacteriodetes. The appearance of this “compositional dysbiosis” was highly correlated in the ASD group with Firmicutes to Bacteriodetes ratio of 31:69 (versus controls 27:73) in the ileal biopsies (*p* < 0.0006) and 32:68 (versus controls 25:75) in the cecal biopsies (*p* < 0.022) (66).

Although microflora are known to alter host immune function, including cytokine production [for review see Ref. (70–73)], to our knowledge there has, to date, been no investigation of the relationship between abnormal microflora and cytokine production in ASD, although a few studies have examined cytokines in ASD patients with bowel symptoms and found positive correlations (72–74).

## Discussion

It is the wide heterogeneity of ASD that poses the greatest challenge. Identifying a common pathophysiology is hampered by such diversity as is the identification of management strategies, both behavioral and medical. Equally those faced with patient care often struggle to discern the range of presentations and the impact this has on management. ASD shares simply, a marked impairment with any of the faculties required for social integration; this can present with a lethargic, disinterested child or an agitated, distracted child or any number of features leading ultimately to impaired social integration. It may be that in order to determine treatment response, we must first delineate/categorize treatable groups. Indeed if some form of environmental insult occurs early on in development (be it infection, toxicant, or other environmental stressor), then it may not only cause variable manifestations based on timing, nature, and genomic individuality, it may also leave no discernible or at least easily discernible trace. It may, as Hertz-Piciotta et al. (54) and Dietert et al. (62) suggest, merely be an event that primes or disrupts a critical window in development. Given the wide-ranging heterogeneity of the disorder and the many faculties required for social integration, it may be neither the insulting agent, the timing, the genomic vulnerability nor the system(s) affected that remain static or, when ASD is taken as a single consequential expression, statistically identifiable.

**Figure 1:**
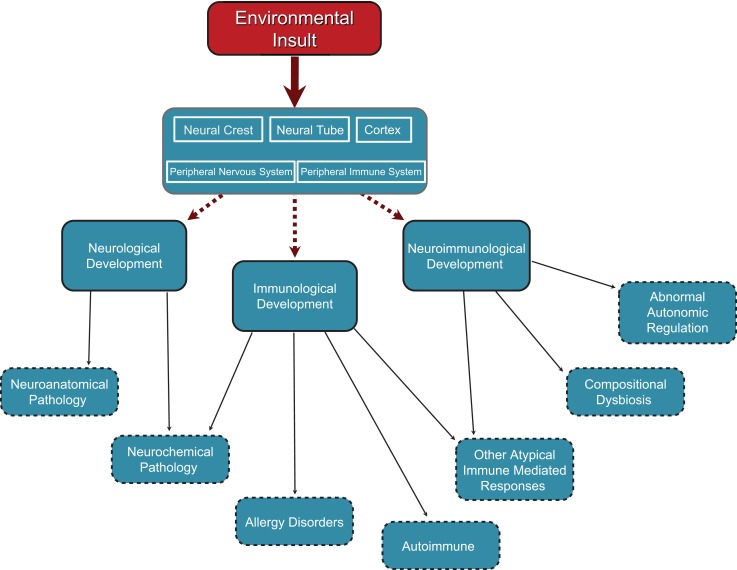
**Cascade of effects originating in neurodevelopmental insults occurring at different points in development**. Insults can occur at any point in neurodevelopment from embryonic through to juvenile and perhaps even adulthood. Insults to neural crest and neural tube development in the embryo would have structural and functional outcomes in all peripheral neural elements including autonomic (sympathetic, parasympathetic), peripheral sensory system, and enteric (gut) nervous system. Neural tube effects might also produce changes in central autonomic functions and/or set points for physiological control systems resident in the hypothalamus and brain stem autonomic nuclei. Factors affecting cortical development would have an impact on higher cortical functions including psychosocial, sensory processing, and motor functioning. Any of these neurodevelopmental effects would have cascading effects through the sensory and neuro-immune pathways to neurological, neuro-immune, and immune functions.

Within the developing neurological, immunological, and neuroimmunological systems, there is vulnerability to environmental insult. Depending on the nature, timing, and duration of insult, neurological, immunological, or neuroimmunological abnormalities may predominate, and the relative proportion each system is affected will vary accordingly (see Figure 1).

Socialization and speech are complex neurological functions. ASD may represent impairment in any system/faculty required to facilitate such complex neurological functions. There is likely ample opportunity for an environmental insult to disrupt one or more of the mechanisms leading to the impairment of the higher-order processes of social integration and along the way disrupt a number of other physiological mechanisms that may contribute or indeed cause the additional and variable behavioral manifestations within the spectrum of disease. As troublesome as the notion may be, and granted there will be reasonable pathophysiological correlations identified particularly within sub-groups, the greatest commonality in ASD may be etiological, and even then it may merely be a trend in human–environment relationship versus an exact noxious substance.

Similar hypotheses have been presented by others: e.g., Dietert et al. (47), Hertz-Picotta et al. (46), Goines and Ashwood (75), and Unwin et al. (76). The central theme is the presence of a variable insult leading to the variable presentation of ASD. In recognition of these previous studies, the hypothesis presented here is referred to as the variable insult model of ASD. Such hypotheses are critical to providing a framework to identify sub-types within the ASD group, building in some predictability both for researchers and for those faced with clinical management. Such sub-typing can be based on the dominant system pathophysiology involved or one can attempt clinical classification. Table 3 is an example of such an attempt at clinical sub-typing based on the variable insult model of ASD.

**Table 3:** **Broad clinical sub-categories of ASD**.

	Prenatal – birth		Infant – early child (birth to 3 years)	
	Early acute insult	Early chronic insult	Late acute insult	Late chronic insult
Congenital abnormalities	++++	+++	+	+
Severe dysmotility	++++	+++	++	++
Sudden regression	+	++	++++	+++
Insidious regression	++	+++	+	++++
Early immune-related issues	+++	++++	++	++
Motor delays	++++	+++	+	+
Family history of autoimmunity	++	++	++++	+++
Gestational exposure	++++	+++	+	+
Early infancy exposure	+	++	++++	++++

*The number of crosses indicates the severity/frequency of each measure relative to age of exposure and its duration. Early acute insult refers to a sudden or relatively sudden, usually marked exposure to xenobiotic, infection, or other environmental stressor during prenatal or gestational periods. Early chronic insult refers to a sustained usually moderate level of exposure over a period of months during prenatal and/or gestational period. Late acute insult refers to a sudden or relatively sudden, usually marked exposure to xenobiotic, infection, or other environmental stressor in infancy to early childhood (birth to 3 years). Late chronic insult refers to a sustained moderate level of exposure over a period of months during infancy to early childhood*.

The sub-categories presented in Table 3 are broad and overlap considerably. More specific sub-typing seems probable, perhaps relating to the intensity of the insult and perhaps more specifically to the offending agent, as suggested recently by Unwin et al. (76). Already abnormal RNA transcription has been identified in ASD children correlating with environmental toxicants versus controls with similar levels of toxicants (77, 78). The transcription abnormalities are specific to the toxicant, raising the possibility of different etiological agents triggering different initial pathophysiological mechanisms sharing only the secondary consequences. The factors involved in the different gene expression in ASD, whether they be linked to genomic individuality, previous exposure, some kind of immunological priming, or abnormal GI flora, raises interesting questions, but in these current considerations the mere difference in RNA transcription between different xenobiotics and also between ASD patients versus control groups raises important questions about accurate delineation of sub-types and the different pathophysiological mechanisms involved in the eventual ASD outcome.

Variable pathophysiological pathways leading to ASD seem likely, and recent advancements in scientific techniques carry the capacity to differentiate each pathway with relevance to the prevention and clinical management of the condition. For example, PCR analysis of GI microflora continues to reveal deeper insights into the common GI abnormalities prevalent in ASD (65, 66, 81). Evidence demonstrates the importance of such microflora on immune and neurological function, and the evolution of GI microflora composition over the first few years of life (82, 83). The compositional dysbiosis discovered by Williams et al. (66) in ASD patients may reflect another common manifestation of ASD due perhaps to similar complexities as is involved in social integration with genomic, neurological, immunological, and neuroimmunological systems required to select and regulate the GI microflora. This may explain the frequently reported presence of abnormal species in ASD and the diversity of such abnormal microflora/pathogens [for review see Ref. (65, 81)]; the selection and regulation processes are also part of the developmental process and are vulnerable to a variety of insults at a variety of levels. ASD diagnosis may be more scientifically sound should it move toward a formulation including environmental exposure, genomic vulnerability, and the identification of the system(s) pathophysiology with treatment interventions based on such measurable criteria. Animal models can serve such ever increasing sub-categorization, modeled to reflect each category and utilized to identify novel therapeutic interventions at specific groups. Without such an appreciation of the variability associated with ASD, it may be difficult to achieve statistical significance in treatment trials targeted at specific deficits.

The variable insult hypothesis predicts diverse immune-related abnormalities, probability of poor GI microflora regulation, variable autonomic function with impaired/disordered autonomic reactivity, and variable neuroanatomical findings. Further, the variable insult model predicts that ASD animal models could be established through a variety of mechanisms. Valproate with an acute single dose administered gestationally at E12.5 leads to ASD symptomatology in rats (79), as does valproate administered in a sub-chronic dosing given between post-natal days P6–12, albeit the sub-chronic dosing may have more sensory issues (80). Unwin et al. (76) proposed that homogeneity may be found by identifying the etiological agent, and presents the differences in associative symptomatology in children with ASD who had either perinatal exposure to selective serotonin re-uptake inhibitor (SSRI) or who had low birth weight (LBW). The SSRI group seemed to have more gastrointestinal disturbance, although it was not clear whether this was a depression effect or drug effect, and the LBW group had more sleep and breathing disturbance. Within the variable insult model of ASD, SSRI exposure may represent greater disruption to the peripheral ANS, perhaps during neural crest formation and development, whereas the LBW group may have suffered more pronounced central autonomic disruption. Difficulties with sensory processing may then affect both groups through different mechanisms with similar social outcomes.

If we start to look at the etiological agents contributing to the development of ASD, then perhaps we can start to find sub-groups. Such an approach brings more complexity to the clinical assessment of patients suffering from ASD but perhaps such a complex condition requires greater effort and more complex formulations prior to predicting prognosis and response to medical or behavioral management. If we go further still and delineate the various pathophysiologies that can lead to ASD symptomatology through animal and human experiments, then early identification of the system(s) requiring attention may be possible and may then better guide harm reduction strategies. Prevention remains the priority, but harm reduction through focused scientific investigation could reduce the burden of disease going forward. For example, in Unwin et al.’s SSRI group, early intervention relating to GI pathology could allow correction of the dysfunctional peripheral autonomic input and thus permit greater sensory integration, and thus improved developmental outcome. Equally addressing the breathing dysrhythmias in the LBW group may improve autonomic reactivity improving both sensory integration and neuro-immune responsiveness. Elsewhere, we present clinical data to support such a possibility through improved autonomic function following management of co-morbid health conditions and the subsequent improvement in ASD symptoms.

## Clinical Application

The pathophysiology of ASD remains largely unknown. Evidence so far suggests prognosis is not pre-determined and there is a dynamic component of the disease. The greater frequency of extra-CNS disease(s) (85–88), increased respiratory and GI-related childhood mortality, and substantially greater risk of progressive seizure disorders (20–22) together with the frequently reported neuroinflammatory changes (2–5) in ASD suggests ongoing disease activity, and whilst targeted treatment trials are awaited these tangible aspects of disease must be the focus of clinical intervention. Actively seeking extra-CNS disease in ASD and pro-actively managing such conditions, with the appreciation that presentation of such disease and response to intervention may not be typical or, whilst the pathophysiology remains ill-defined, predictable, is a pillar of harm reduction. Delineating sub-groups further will hopefully help predict the areas requiring further attention in each patient group.

## Summary

Autism spectrum disorder is a severe neurological condition with variable presentation, disease evolution, and variable, albeit generally poor, functional outcomes. Patients with ASD have greater risk of physical and mental health complications, and also a greater mortality. Neuroinflammation, peripheral immune abnormalities, and environmental factors have consistently been identified, further supporting the need for research that prioritizes disease prevention and harm reduction. The results from large epidemiological studies are awaited to identify potential key areas for this research.

Heterogeneity has been a significant barrier to successful intervention in ASD. It may be that the commonality of impaired social integration represents dysfunction of a wide variety of systems and faculties during a crucial developmental period required for the complexities of social integration and as such the commonality is merely etiological. Similar levels of intricacy may explain the propensity to abnormal acquisition of GI flora during an equally important microbiota developmental period. Here, we reviewed the most promising research findings to explain the diverse neurological, immunological, and neuroimmunological abnormalities in ASD within consideration of variable environmental insults. In our opinion, this provides a useful framework to understand and further explore this devastating neurodevelopmental condition.

## Conflict of Interest Statement

The Guest Associate Editor Adam Denes declares that, he holds a permanent position at the Institute of Experimental Medicine, Budapest, Hungary, and despite being a visiting scientist at the same institution as authors Daniel K. Goyal and Jaleel A. Miyan, the review process was handled objectively and no conflict of interest exists.
